# Resilience and self-rated health among Ukrainian war-displaced adults in Poland: a cross-sectional study

**DOI:** 10.3389/fpubh.2026.1830164

**Published:** 2026-05-29

**Authors:** Małgorzata Szkup, Paulina Jeż, Marta Kożybska, Sara Szalaty-Domaradzka, Paulina Zabielska

**Affiliations:** 1 Subdepartment of Social Nursing, Department of Health Sciences, Pomeranian Medical University in Szczecin, Szczecin, Poland; 2 Subdepartment of Medical Law, Department of Social Medicine, Pomeranian Medical University in Szczecin, Szczecin, Poland; 3 Subdepartment of Social Medicine and Public Health, Department of Social Medicine, Pomeranian Medical University in Szczecin, Szczecin, Poland

**Keywords:** migration and health, public health, refugee health, resilience, self-perceived health, Ukrainian refugees, war-displaced populations

## Abstract

**Introduction:**

War-related displacement creates major public health challenges, yet the relationship between resilience and broader health functioning in refugee populations remains insufficiently understood. This study examined the association between resilience and self-rated health among Ukrainian war-displaced adults living in Poland.

**Methods:**

A cross-sectional observational study was conducted in 2024 among 390 Ukrainian adults residing in Poland who had experienced war-related displacement. Data were collected using a paper-based survey including the Resilience Scale and an author-designed questionnaire covering self-rated health, physical fitness, physical activity, wellbeing, and self-reported health problems, with selected measures assessed for both the period in Ukraine and the current period in Poland. Associations between resilience level and health-related variables were analyzed using chi-square and Fisher's exact tests.

**Results:**

Of the 390 participants, 46.67% were classified as having moderately high to high resilience, 29.74% as having moderately low to moderate resilience, and 23.59% as having very low to low resilience. More than half of the respondents reported no health problems (53.33%). Participants without health problems were more likely to have moderately high to high resilience than those reporting health problems (54.33% vs. 37.91%; *p* = 0.003). Resilience was significantly associated with respiratory disorders (*p* = 0.041), nervous system disorders (*p* = 0.006), gastrointestinal disorders (*p* < 0.001), and musculoskeletal disorders (*p* = 0.046), but not with cardiovascular disorders (*p* = 0.502). Higher resilience was also significantly associated with more favorable self-rated health, physical functioning, physical activity, and wellbeing, both retrospectively for the period in Ukraine and currently in Poland. For all four domains, associations were statistically significant for both reference periods, with p-values ranging from *p* = 0.005 to *p* < 0.001. Effect-size estimates were small to small-to-moderate.

**Conclusion:**

Resilience appears to be a relevant public health construct among war-displaced populations and may help identify individuals at greater risk of poorer health functioning. Strengthening adaptive resources should therefore be considered as part of comprehensive health and psychosocial support for refugees.

## 1 Introduction

Contemporary migration is becoming increasingly complex and intensified, driven primarily by armed conflicts, economic inequalities, and environmental crises. Forced migration is a particularly important component of this process, as it can rapidly reshape the demographic structure of receiving countries and place substantial pressure on public health systems (1). In this context, Poland provides a notable example of a country that, within a relatively short period, has shifted from being predominantly a country of emigration to becoming one of the key receiving countries in Central and Eastern Europe (2).

The growing role of Poland as a destination country has been especially evident in relation to migration from Ukraine. After 2014, migration to Poland increased markedly, initially taking largely economic and circular forms (3). A major turning point occurred on 24 February 2022, when Russia launched its full-scale invasion of Ukraine, triggering the largest refugee crisis in Europe in decades. According to analyses conducted by the Centre of Migration Research at the University of Warsaw, nearly 3 million war refugees crossed the Polish–Ukrainian border during the first 2 months of the war alone (4). Data from the Office for Foreigners further indicate that Ukrainian citizens currently constitute the largest group of foreign nationals residing in Poland, with approximately 1.55 million individuals holding valid residence permits (5).

This large-scale forced migration has consequences not only for population structure, but also for health. Individuals fleeing war are exposed to multiple pre-migration stressors, including direct threats to life, violence, the loss of loved ones, and the destruction of their previous living environment. These experiences are often followed by post-migration stressors, such as legal uncertainty, language barriers, economic hardship, limited access to healthcare, and weakened social support networks (1, 6). The cumulative burden of these adversities has been associated with an increased risk of mental health problems, reduced wellbeing, and more severe somatic complaints (1, 6).

One of the constructs most frequently examined in research on adaptation to adversity is resilience. It is commonly defined as a process of relatively positive adaptation despite exposure to severe stressors, trauma, or other forms of adversity (7). At the same time, the concept remains heterogeneous, and has been variously conceptualized as a trait, a process, a resource, or an outcome of adaptation (7, 8). This multidimensionality has made resilience an increasingly important concept in the health sciences, particularly in studies of individuals exposed to chronic stress, war-related trauma, forced migration, and prolonged uncertainty (7–9).

In refugee populations, resilience has been studied primarily in relation to mental health. Systematic reviews focusing on Ukrainian refugees and internally displaced persons indicate that the literature has largely concentrated on psychological risk factors, such as anxiety, depression, and post-traumatic symptoms, whereas less attention has been paid to protective factors and their relationship with broader dimensions of health (9). This gap is important because resilience may function as an adaptive resource not only in the emotional domain, but also in everyday functioning and in coping with health-related burdens.

An important but still insufficiently explored issue is the relationship between resilience and the somatic dimension of health, particularly in the context of forced migration. Much of the existing literature has focused on psychological adaptation, while the association between general health status and resilience has received far less attention. At the same time, self-rated health (SRH) is one of the most widely used indicators in epidemiology and public health, and its prognostic value has been repeatedly demonstrated, including in relation to mortality and long-term functioning (10). A better understanding of the relationship between resilience and self-rated health may therefore help identify individuals who are particularly vulnerable to adaptation difficulties and inform the development of more appropriate psychosocial and health interventions.

In the context of the present study, a brief author-designed health-related questionnaire was used to capture several broad domains of subjective health-related functioning. This approach was chosen to obtain a concise and feasible assessment suitable for a paper-based survey among Ukrainian war-displaced adults recruited in community settings. The questionnaire was not intended to replace standardized clinical or psychometric instruments, but rather to provide context-sensitive self-report indicators of overall health, physical functioning, physical activity, wellbeing, and selected self-reported health problems. This was considered particularly relevant because displaced populations may experience overlapping physical, psychological, and functional burdens, while lengthy assessment batteries may increase respondent burden and reduce feasibility in field-based data collection.

Against this background, further investigation of the relationship between resilience and self-rated health among adults forcibly displaced from Ukraine and currently residing in Poland is warranted. In the present study, self-rated health is understood as a global, subjective assessment of health that may integrate physical, psychological, and functional dimensions of wellbeing. This makes SRH particularly relevant in refugee health research, where objective clinical data may be difficult to obtain and where subjective health appraisal can reflect the combined effects of war-related experiences, post-migration stressors, and current living conditions. In addition to global SRH, selected self-reported somatic health problems were considered as complementary indicators of health-related burden. A more comprehensive understanding of these associations may have important implications for public health practice, particularly for planning support for refugee populations and developing interventions aimed at strengthening adaptive capacity in the context of a prolonged migration crisis (1, 6, 9).

Given the limited evidence on the relationship between resilience and global self-rated health among forcibly displaced populations, this study aimed to assess the association between resilience and self-rated health among Ukrainian war-displaced adults residing in Poland. Selected self-reported somatic health problems were additionally analyzed to provide a broader context for interpreting health-related burden in this population and to inform public health and psychosocial support strategies.

## 2 Material and methods

### 2.1 Study design

A cross-sectional observational design was adopted for this study, using a descriptive and correlational analytical framework. Because the study was non-experimental in nature, no intervention was introduced and no variables were manipulated. Instead, the analysis focused on naturally occurring associations between the variables of interest. The conduct and reporting of the study were guided by the STROBE (Strengthening the Reporting of Observational Studies in Epidemiology) recommendations to ensure clarity, transparency, and methodological robustness.

### 2.2 Participants and eligibility criteria

The study was conducted in 2024 among adults affected by the war in Ukraine. Eligibility for participation was restricted to individuals who were 18 years of age or older, held Ukrainian citizenship, and were residing in Poland at the time of the survey. In addition, participants were required to meet at least one war-related displacement criterion: either having left Ukraine because of threats arising from military actions, or having resided in Poland before 24 February 2022 and being unable or unwilling to return to Ukraine due to safety concerns related to the armed conflict. Thus, war-related displacement was incorporated into the study as an eligibility criterion and contextual characteristic of the sample. However, individual exposure to war-related events, trauma severity, and perceived war-related stress were not assessed using separate standardized measures and were not included as independent variables or covariates in the analyses. To ensure the reliability of self-administered data collection, only individuals with sufficient written proficiency in Ukrainian to complete the questionnaire independently were included. All participants provided written informed consent before participation. Participants were excluded if they declined to provide informed consent, did not satisfy the inclusion criteria, or returned incomplete survey forms.

### 2.3 Sample size

Sample size considerations were based on population statistics from the Polish Office for Foreigners, according to which approximately 1.55 million Ukrainian citizens held valid residence permits in Poland as of 24 February 2025. The minimum sample size was estimated using OpenEpi, version 3.01, with a 95% confidence level, a 5% margin of error, and a conservative assumed population proportion of 50%. Under these assumptions, the required sample size was 385 participants. This calculation was intended to provide a conservative population-based estimate of the minimum sample size rather than an a priori power calculation for hypothesis testing. Because of the exploratory cross-sectional design of the study and the limited availability of prior evidence on expected effect sizes for associations between resilience and self-rated health in Ukrainian war-displaced adults, no effect-size-based sample size calculation was performed. The achieved sample of 390 respondents slightly exceeded the population-based estimate and was considered adequate for the exploratory analyses, although the interpretation of association tests should take this limitation into account.

### 2.4 Data collection procedure

Data collection was carried out using a structured survey administered in paper-and-pencil format. A non-probability convenience sampling strategy was applied. Participants were recruited among Ukrainian adults residing in Poland who met the eligibility criteria for the study. Recruitment was conducted in settings accessible to Ukrainian migrants and war-displaced persons, including community support centres, non-governmental organizations providing assistance to Ukrainian refugees, educational institutions, workplaces, and informal Ukrainian support networks. Potential participants were informed about the study by members of the research team or persons involved in questionnaire distribution and were invited to participate if they met the inclusion criteria. No random sampling frame was available, and recruitment depended on participants' accessibility and willingness to take part in the study. The study package consisted of standardized psychometric instrument (Resilience Scale—RS-25) and a sociodemographic questionnaire designed specifically for the purposes of the present study. All questionnaires were provided in Ukrainian.

Prior to enrolment, potential participants received written information outlining the aims of the study, the principles of confidentiality and anonymity, and their right to withdraw from participation at any time without negative consequences. Participation was entirely voluntary, and no financial or non-financial compensation was provided.

The study protocol was approved by the Bioethics Committee of the [information removed for anonymized review process] (Approval No. KB.006.41.2024).

In total, 500 questionnaire sets were distributed, and 390 completed sets were returned, yielding a response rate of 78%. Because the study was anonymous, the research team had access only to completed questionnaires and was unable to identify or follow up individuals at earlier stages of recruitment or response.

### 2.5 Data collection instruments

Study data were obtained using an author-designed survey together with a standardized psychometric measure. The author-designed questionnaire was developed specifically for the purposes of this exploratory cross-sectional study to collect sociodemographic data, information on self-reported health problems, and subjective assessments of health-related functioning. The content of the questionnaire was prepared by the research team on the basis of the study objectives and the need to capture brief, comprehensible self-report indicators relevant to the situation of Ukrainian war-displaced adults. Before data collection, the questionnaire was reviewed by the research team for content relevance, clarity, linguistic comprehensibility, and suitability for self-administration in paper-and-pencil format. The health-related domains were intended as broad subjective self-assessments rather than standardized clinical or psychometric constructs. Therefore, simple wording was used to minimize respondent burden and reduce the risk of misunderstanding. In addition, a brief pilot test was conducted among Ukrainian-speaking adults to assess the comprehensibility, acceptability, and feasibility of completing the questionnaire, including whether the health-related domains and the response scale were understood as intended. Based on the pilot testing, minor wording adjustments were introduced before the final version was administered. Nevertheless, some variability in respondents' individual interpretation of these broad domains cannot be excluded. The questionnaire was not intended to function as a standardized psychometric scale, and no formal psychometric validation was performed. The author-designed questionnaire, including the original Ukrainian version administered to participants and an English translation for review and transparency purposes, is provided as Supplementary Appendix 1.

The questionnaire included sociodemographic variables, including age, gender, and marital status, as well as questions addressing major health problems and self-rated health in several domains. Regarding major health problems, participants were asked to indicate whether they had disorders affecting the circulatory, respiratory, nervous, gastrointestinal, and musculoskeletal systems. This was a multiple-choice item, and participants could indicate more than one health problem. Therefore, the health-problem categories were not mutually exclusive. Participants indicated for each group whether a disorder was present or not. Self-rated health-related functioning was assessed in four domains: overall health, physical functioning, physical activity, and wellbeing. Overall health referred to the participant's global subjective appraisal of general health. Physical functioning referred to perceived ability to perform everyday physical activities and bodily functioning. Physical activity referred to the participant's subjective assessment of their level of movement or activity in daily life. Wellbeing referred to perceived subjective wellbeing in daily life. Each domain was rated on a five-point ordinal scale: very poor, poor, fair, good, and very good. To reflect the migration context, selected health-related assessments were obtained for two time points: retrospectively for the period prior to migration, when respondents were living in Ukraine, and for the current period of residence in Poland. The pre-migration assessment was therefore retrospective and reflected participants' current recollection of their health-related functioning before displacement.

Resilience was measured using the Resilience Scale−25 (RS-25), a widely used self-report instrument developed by Wagnild and Young (11). In this instrument, resilience is understood as a positive personal resource that facilitates adaptation to adversity and supports positive psychological functioning despite stress or difficult life circumstances. The RS-25 conceptualizes resilience through five core dimensions: purpose, equanimity, self-reliance, perseverance, and acceptance of self and life. The scale is intended to capture individual differences in resilience-related psychological resources rather than to serve as a diagnostic tool.

According to written information received from the authors of the RS-25, no Ukrainian version of the scale was available. Formal permission to translate and administer the instrument in Ukrainian was obtained prior to data collection. The Ukrainian-language version was prepared using a translation and back-translation procedure. First, the RS-25 was translated independently into Ukrainian by two translators. The two translated versions were then compared and reconciled into a single Ukrainian version. This version was subsequently back-translated into English and compared with the original scale to assess semantic consistency and conceptual equivalence. Any discrepancies were discussed and resolved before the questionnaire was administered. This procedure focused on linguistic clarity and conceptual equivalence between the original and Ukrainian versions; however, it did not constitute a full psychometric validation of the Ukrainian version, and no adaptation-specific statistical indicators were calculated.

The RS-25 consists of 25 items rated on a 7-point Likert scale. Total scores range from 25 to 175, with higher scores reflecting higher levels of resilience. Based on the original RS-25 score interpretation proposed by Wagnild (12), we combined adjacent categories for reporting purposes: very low to low resilience (25–100 points), moderately low to moderate resilience (101–130 points), or moderately high to high resilience (131–175 points).

Previous studies have shown that the RS-25 has good psychometric properties, including high internal consistency and satisfactory temporal stability. Its validity has also been supported through associations with indicators of psychological wellbeing and adaptive coping. Given these characteristics, the instrument was considered suitable for the assessment of resilience in the present sample.

### 2.6 Data analysis

All statistical analyses were conducted using Microsoft Excel 2019 and Statistica 13 (TIBCO Software Inc.). The final analysis included 390 fully completed questionnaires. Descriptive statistics were calculated for all study variables. Continuous data were summarized using the mean, standard deviation, median, quartiles, and minimum–maximum range, whereas categorical variables were described using frequencies and percentages.

The analytical part of the study focused primarily on the relationship between resilience level and selected health-related variables. Associations between categorical variables were assessed using the chi-square test. Whenever the assumptions for the chi-square approximation were not satisfied, Fisher's exact test was used instead. To complement statistical significance testing, Cramér's V was calculated as a measure of effect size for associations between categorical variables. Effect-size values were interpreted descriptively, with higher values indicating stronger associations. The analyses were exploratory and bivariate in nature. No multivariable adjustment was performed; therefore, the results should be interpreted as unadjusted associations rather than independent effects. This approach was chosen to describe initial patterns of association between resilience levels and health-related variables in the study population.

This analytical strategy was applied to examine differences in the distribution of resilience levels according to the presence of reported health problems and to assess associations between resilience and self-rated health, physical fitness, physical activity, and wellbeing. For self-rated measures, analyses were performed separately for two reference periods: retrospectively for the time when respondents were living in Ukraine and for their current situation in Poland. A significance level of 0.05 was adopted throughout, and *p-values* below this threshold were considered statistically significant.

## 3 Results

Table 1 presents the sociodemographic and health-related characteristics of the study group. The sample included 390 participants and was predominantly female (72.56%; *n* = 283). The mean age was 38.61 years (SD = 12.88), while the median age was 38 years (IQR: 29–47; range: 18–78). Almost half of the respondents had tertiary education (46.67%; *n* = 182). In terms of relationship status, 43.08% (*n* = 168) were in a relationship with a partner living in Poland, 16.41% (*n* = 64) had a partner residing in Ukraine, and 40.26% (*n* = 157) were single. More than half of the participants declared no health problems (53.33%; *n* = 208). Among the reported conditions, the most common were disorders of the nervous system (16.41%; *n* = 64), musculoskeletal disorders (15.38%; *n* = 60), gastrointestinal disorders (13.59%; *n* = 53), and circulatory disorders (13.33%; *n* = 52), whereas mental health problems were indicated by 11.03% of respondents (*n* = 43). Because health problems were assessed using a multiple-choice item, these categories were not mutually exclusive, and some participants could report more than one health problem. The number and specific combinations of co-occurring health problems were not analyzed as separate variables. According to the RS-25 classification, 46.67% of participants (*n* = 182) had moderately high to high resilience, 29.74% (*n* = 116) had moderately low to moderate resilience, and 23.59% (*n* = 92) were classified as having very low to low resilience (Table 1). Table 2 presents the distribution of resilience levels (RS-25) according to the presence of selected self-reported health problems in the study group. Health status was assessed on the basis of participants' reports of disorders affecting the circulatory, respiratory, nervous, gastrointestinal, and musculoskeletal systems, as well as the absence of any reported health problems (Table 1).

**Table 1:** Characteristics of the study group.

Characteristic		Total (*N* = 390)
Sex	Female	283 (72.56%)
	Male	104 (26.67%)
	Non-binary	2 (0.51%)
	Prefer not to say	1 (0.26%)
Age [years]	Mean (SD)	38.61 (12.88)
	Median (quartiles)	38 (29–47)
	Range	18–78
Education level	Primary education	13 (3.33%)
	Vocational education	66 (16.92%)
	Secondary education	129 (33.08%)
	Higher education	182 (46.67%)
Marital status	In a relationship. partner living in Poland	168 (43.08%)
	In a relationship. partner living in Ukraine	64 (16.41%)
	Single	157 (40.26%)
	No response	1 (0.26%)
Main health problem^*^	I do not have any health problems	208 (53.33%)
	Cardiovascular disorders	52 (13.33%)
	Respiratory disorders	34 (8.72%)
	Nervous system disorders	64 (16.41%)
	Digestive system disorders	53 (13.59%)
	Musculoskeletal disorders	60 (15.38%)
	Mental health problems	43 (11.03%)
**RS-25**	***n***	**%**
Very low to low total RS-25 score	92	23.59%
Moderately low to moderate total RS-25 score	116	29.74%
Moderately high to high total RS-25 score	182	46.67%

^*^Multiple-choice question; percentages may not sum to 100.

**Table 2:** Analysis of differences between resilience levels and the prevalence of health problems among Ukrainian refugees.

Total RS-25 score	No health problems		p/Cramér's V
	Yes (*N* = 208)	No (*N* = 182)	
Very low to low	38 (18.27%)	54 (29.67%)	***p*** **=** **0.003 0.174**
Moderately low to moderate	57 (27.40%)	59 (32.42%)	
Moderately high to high	113 (54.33%)	69 (37.91%)	
	**Cardiovascular disorders**		**p/Cramér's V**
	**Yes (*****N*** **=** **52)**	**No (*****N*** **=** **338)**	
Very low to low	9 (17.31%)	83 (24.56%)	*p* = 0.502
Moderately low to moderate	16 (30.77%)	100 (29.59%)	0.059
Moderately high to high	27 (51.92%)	155 (45.86%)	
	**Respiratory disorders**		**p/Cramér's V**
	**Yes (*****N*** **=** **34)**	**No (*****N*** **=** **356)**	
Very low to low	14 (41.18%)	78 (21.91%)	***p*** **=** **0.041**
Moderately low to moderate	8 (23.53%)	108 (30.34%)	0.128
Moderately high to high	12 (35.29%)	170 (47.75%)	
	**Nervous system disorders**		**p/Cramér's V**
	**Yes (*****N*** **=** **64)**	**No (*****N*** **=** **326)**	
Very low to low	25 (39.06%)	67 (20.55%)	***p*** **=** **0.006**
Moderately low to moderate	16 (25.00%)	100 (30.67%)	0.162
Moderately high to high	23 (35.94%)	159 (48.77%)	
	**Digestive system disorders**		**p/Cramér's V**
	**Yes (*****N*** **=** **53)**	**No (*****N*** **=** **337)**	
Very low to low	23 (43.40%)	69 (20.47%)	***p*** **<** **0.001**
Moderately low to moderate	17 (32.08%)	99 (29.38%)	0.207
Moderately high to high	13 (24.53%)	169 (50.15%)	
	**Musculoskeletal disorders**		**p/Cramér's V**
	**Yes (*****N*** **=** **60)**	**No (*****N*** **=** **330)**	
Very low to low	15 (25.00%)	77 (23.33%)	***p*** **=** **0.046**
Moderately low to moderate	25 (41.67%)	91 (27.58%)	**0.126**
Moderately high to high	20 (33.33%)	162 (49.09%)	

p-values were calculated using the chi-square test or Fisher's exact test, as appropriate. Cramér's V was calculated as a measure of effect size for categorical associations.

The distribution of resilience levels differed significantly between participants who reported no health problems and those who reported at least one health problem (*p* = 0.003; Cramér's V = 0.174). Among participants without health problems, 54.33% were classified as having moderately high to high resilience, compared with 37.91% among those reporting health problems. Statistically significant differences in the distribution of resilience levels were also found according to the presence of respiratory disorders (*p* = 0.041; Cramér's V = 0.128), nervous system disorders (*p* = 0.006; Cramér's V = 0.162), gastrointestinal disorders (*p* < 0.001; Cramér's V = 0.207), and musculoskeletal disorders (*p* = 0.046; Cramér's V = 0.126). No statistically significant difference was found for cardiovascular disorders (*p* = 0.502; Cramér's V = 0.059) (Table 2).

Table 3 presents the distribution of RS-25 resilience levels across categories of self-rated health, physical functioning, physical activity, and wellbeing, separately for two reference periods: retrospectively for the period formerly spent in Ukraine and currently in Poland.

**Table 3:** Distribution of resilience levels across self-rated health-related domains in two reference periods: formerly in Ukraine and currently in Poland.

Level of resilience	Formerly in Ukraine						Currently in Poland					
	Very poor *N* = 13 (%)	Poor *N* = 11 (%)	Fair *N* = 39 (%)	Good *N* = 131 (%)	Very good *N* = 192 (%)	p/CV	Very poor *N* = 9 (%)	Poor *N* = 20 (%)	Fair *N* = 95 (%)	Good *N* = 123 (%)	Very good *N* = 140 (%)	p/CV
	Overall health											
Very low to low	5 (38.46)	4 (36.36)	17 (43.59)	30 (22.90)	36 (18.75)	**<0.001** **0.206**	2 (22.22)	8 (40.0)	31 (32.63)	31 (25.20)	20 (14.29)	**<0.001** ** 0.242**
Moderately low to moderate	2 (15.38)	2 (18.18)	10 (25.64)	56 (42.75)	46 (23.96)		2 (22.22)	8 (40.0)	37 (38.95)	43 (34.96)	26 (18.57)	
Moderately high to high	6 (46.15)	5 (45.45)	12 (30.77)	45 (34.35)	110 (57.29)		5 (55.56)	4 (20.0)	27 (28.42)	49 (39.84)	94 (67.14)	
	Physical functioning											
Very low to low	5 (41.67)	3 (27.27)	14 (41.18)	41 (30.60)	29 (14.87)	***p*** **=** **0.001 0.187**	4 (30.77)	7 (33.33)	27 (32.53)	38 (32.48)	16 (10.48)	***p*** **<** **0.001 0.248**
Moderately low to moderate	2 (16.67)	3 (27.27)	8 (23.53)	47 (35.07)	56 (28.72)		4 (30.77)	8 (38.10)	32 (38.55)	37 (31.62)	35 (22.88)	
Moderately high to high	5 (41.67)	5 (45.45)	12 (35.29)	46 (34.33)	110 (56.41)		5 (38.46)	6 (28.57)	24 (28.92)	42 (35.90)	102 (66.67)	
	Physical activity											
Very low to low	6 (46.15)	4 (40.00)	12 (23.53)	37 (33.64)	33 (16.34)	***p*** **<** **0.001 0.192**	6 (35.29)	8 (27.59)	26 (33.33)	34 (31.19)	17 (11.11)	***p*** **<** **0.001 0.253**
Moderately low to moderate	3 (23.08)	1 (10.00)	17 (33.33)	40 (36.36)	55 (27.23)		7 (41.18)	13 (44.83)	28 (35.90)	35 (32.11)	33 (21.57)	
Moderately high to high	4 (30.77)	5 (50.00)	22 (43.14)	33 (30.00)	114 (56.44)		4 (23.53)	8 (27.59)	24 (30.77)	40 (36.70)	103 (67.32)	
	Wellbeing											
Very low to low	4 (30.77)	5 (45.45)	10 (31.25)	32 (26.23)	41 (19.71)	***p*** **=** **0.005 0.169**	2 (20.00)	17 (60.71)	28 (30.43)	33 (23.24)	12 (10.34)	***p*** **<** **0.001 0.290**
Moderately low to moderate	3 (23.08)	3 (27.27)	9 (28.12)	50 (40.98)	51 (24.52)		4 (40.00)	8 (28.57)	34 (36.96)	50 (35.21)	20 (17.24)	
Moderately high to high	6 (46.15)	3 (27.27)	13 (40.62)	40 (32.79)	116 (55.77)		4 (40.00)	3 (10.71)	30 (32.61)	59 (41.55)	84 (72.41)	

The table presents the distribution of RS-25 resilience levels across categories of self-rated overall health, physical functioning, physical activity, and wellbeing. Each domain is shown separately for two reference periods: the retrospective assessment of the period formerly spent in Ukraine and the current assessment in Poland. Percentages are calculated within each response category. p-values were calculated using Fisher's exact test. Cramér's V was calculated as a measure of effect size for categorical associations.

For self-rated health, among participants who rated their health as very good, 57.29% for the period in Ukraine and 67.14% for the current period in Poland were classified as having moderately high to high resilience. Among participants who rated their health as fair, the corresponding proportions were 30.77% for Ukraine and 28.42% for Poland. The association between resilience level and self-rated health was statistically significant for both reference periods, with small-to-moderate effect-size estimates (Ukraine: *p* < 0.001, Cramér's V = 0.206; Poland: *p* < 0.001, Cramér's V = 0.242).

For physical functioning, among participants who rated their functioning as very good, 56.41% for the period in Ukraine and 66.67% for the current period in Poland were classified as having moderately high to high resilience. Among participants who rated their physical functioning as fair, the corresponding proportions were 35.29% for Ukraine and 28.92% for Poland. The association between resilience level and physical functioning was statistically significant for both reference periods, with small-to-moderate effect-size estimates (Ukraine: *p* = 0.001, Cramér's V = 0.187; Poland: *p* < 0.001, Cramér's V = 0.248).

For physical activity, among participants who rated their activity as very good, 56.44% for the period in Ukraine and 67.32% for the current period in Poland were classified as having moderately high to high resilience. Among participants who rated their physical activity as fair, the corresponding proportions were 43.14% for Ukraine and 30.77% for Poland. The association between resilience level and physical activity was statistically significant for both reference periods, with small-to-moderate effect-size estimates (Ukraine: *p* < 0.001, Cramér's V = 0.192; Poland: *p* < 0.001, Cramér's V = 0.253).

For subjective wellbeing, among participants who rated their wellbeing as very good, 55.77% for the period in Ukraine and 72.41% for the current period in Poland were classified as having moderately high to high resilience. Among participants who rated their wellbeing as fair, the corresponding proportions were 40.62% for Ukraine and 32.61% for Poland. The association between resilience level and subjective wellbeing was statistically significant for both reference periods, with small-to-moderate effect-size estimates (Ukraine: *p* = 0.005, Cramér's V = 0.169; Poland: *p* < 0.001, Cramér's V = 0.290) (Table 3).

## 4 Discussion

This study examined unadjusted associations between resilience and health-related variables among Ukrainian war-displaced adults residing in Poland. Higher resilience levels were observed more frequently among participants reporting more favorable self-rated health, physical fitness, physical activity, and subjective wellbeing. However, the corresponding effect-size estimates were generally small to small-to-moderate, indicating that the observed associations should be interpreted with appropriate caution. In addition, respondents who reported no health problems were more likely to exhibit higher resilience than those reporting at least one health problem. Differences in the distribution of resilience levels were also observed across several categories of self-reported somatic morbidity, particularly respiratory, nervous system, gastrointestinal, and musculoskeletal disorders. Taken together, these findings suggest that resilience may be a relevant correlate of broader health functioning in forcibly displaced populations, although the exploratory and unadjusted nature of the analyses should be taken into account.

Our results are consistent with previous evidence showing that resilience is related to adaptation and health-related functioning in refugee and war-affected populations. A recent systematic review of Ukrainian refugees and internally displaced persons showed that resilience is shaped by both individual and environmental factors, including coping strategies, social support, community belonging, and opportunities for meaningful adaptation in the host country. Importantly, that review also noted that the literature has focused predominantly on psychological symptoms and far less on the relationship between resilience and broader health indicators. In this context, the present study contributes to the field by showing that resilience is associated not only with subjective wellbeing, but also with self-rated general health and selected somatic complaints. This broader perspective is particularly relevant in refugee health research, where mental and physical burdens frequently coexist and interact (9, 13).

The observed association between lower resilience and the presence of respiratory, gastrointestinal, nervous system, and musculoskeletal problems may be considered in light of a stress-related and biopsychosocial framework. Forced migration is typically preceded by exposure to war-related trauma and followed by post-migration stressors such as uncertainty, family separation, financial strain, disrupted routines, and barriers to healthcare. One possible explanation is that these exposures may contribute simultaneously to poorer physical health and reduced adaptive capacity. Another possible hypothesis is that poorer health may limit the psychological and behavioral resources needed for effective coping. However, because of the cross-sectional design and unadjusted analyses, the present study cannot determine the direction of these relationships or test these mechanisms directly. This interpretation is compatible with current evidence showing that post-migration stressors are closely linked to both psychological distress and somatic symptoms, and that refugees commonly experience overlapping mental and physical health needs rather than isolated disorders (14–16).

An important finding of the present study is that resilience level differed significantly across categories of self-rated health. Self-rated health is widely used in public health because it captures an individual's integrated perception of physical, psychological, and functional status and has repeatedly been shown to predict morbidity and mortality. Our findings are consistent with the interpretation that resilience may be linked to a person's broader sense of health and functioning, although this relationship should be interpreted as unadjusted and non-causal. This interpretation is in line with previous studies showing a positive relationship between resilience and self-rated health in adult populations, including longitudinal evidence suggesting that resilience and self-rated health may reinforce one another over time. In displaced populations, a bidirectional relationship between adaptive capacity and perceived health may therefore be a plausible hypothesis. However, the present cross-sectional data do not allow temporal ordering or reciprocal effects to be tested directly (17–20).

The pattern observed for physical fitness and physical activity also deserves attention. Participants with higher resilience were more likely to rate their physical fitness and activity levels favorably, both retrospectively for the period in Ukraine and currently in Poland. Although the cross-sectional design precludes causal inference, these findings may be considered in light of previous research suggesting reciprocal links between physical activity, self-rated health, and resilience. One possible hypothesis is that physical activity may support resilience through behavioral activation, self-efficacy, physiological stress regulation, and preservation of daily structure. Conversely, higher resilience may facilitate engagement in health-promoting behaviors even under adverse living conditions. These possible pathways should be tested in future longitudinal or intervention studies. In refugee populations, these mechanisms may be especially important because displacement often disrupts routine, mobility, social participation, and access to safe spaces for exercise (19, 21, 22).

The finding that respondents with higher resilience reported better wellbeing is also consistent with previous work on Ukrainian refugees in Poland. Studies conducted after the 2022 escalation of the war documented high levels of distress, anxiety, depressive symptoms, and post-traumatic stress among Ukrainian refugees, while also indicating that coping resources and psychosocial capital remain important for adaptation. Our results complement this literature by suggesting that resilience may differentiate not only emotional adjustment but also broader health self-appraisal. This is important from a public health perspective, because refugee support systems are often organized around acute material needs and mental health symptoms, while less attention is paid to positive resources that may shape longer-term adaptation (23–25).

These findings may have several practical implications. In exploratory settings, resilience may be considered alongside brief self-rated health measures when identifying displaced adults who may require additional health or psychosocial support. The results also suggest that support for refugees should extend beyond symptom reduction and consider resources that may facilitate adaptation, such as social support, agency, daily routine, language-facilitated access to services, and opportunities for meaningful activity. Given that the associations observed in this study also involve physical fitness and physical activity, resilience-informed support may be valuable when integrated with broader health promotion strategies rather than restricted to psychological care alone. Community-based and culturally sensitive approaches may be particularly valuable in this respect (9, 25).

The findings should be interpreted in light of several limitations. Due to the cross-sectional design, no causal conclusions can be drawn, and it remains uncertain whether higher resilience contributes to better health perception and functioning, whether better health supports resilience, or whether both are influenced by shared underlying determinants. Consequently, any discussion of potential mechanisms or bidirectional relationships should be interpreted as hypothesis-generating rather than confirmatory. Furthermore, part of the health assessment was based on retrospective reports referring to the period before displacement. These retrospective ratings may have been affected by recall bias and may also have been influenced by respondents' current health status, wellbeing, and post-migration experiences in Poland. Therefore, comparisons between the former situation in Ukraine and the current situation in Poland should not be interpreted as equivalent to prospectively collected longitudinal data, but rather as subjective retrospective appraisals that may reflect both past experiences and current circumstances. The reliance on self-reported health problems and self-rated measures also raises the possibility of common-method bias. Although participants could report more than one health problem, comorbidity burden and specific combinations of co-occurring conditions were not analyzed as separate variables. Therefore, the observed associations between resilience and individual health-problem categories may partly reflect the influence of overlapping health problems. In addition, the author-designed questionnaire used to assess self-rated health domains and self-reported health problems was developed for the purposes of this study and was not formally psychometrically validated. Although the questionnaire was reviewed by the research team and pilot-tested for clarity, comprehensibility, acceptability, feasibility, and understanding of the health-related domains before data collection, the lack of formal validation may limit the precision and comparability of these measures. In particular, some variability in how respondents interpreted broad domains such as physical functioning or wellbeing cannot be excluded. In particular, the broad self-rated domains should be interpreted as subjective indicators of health-related functioning rather than as standardized clinical measures. Another limitation concerns the language version of the RS-25. Although the Ukrainian version was prepared using a translation and back-translation procedure and reviewed for semantic consistency, conceptual equivalence, and comprehensibility, a full formal psychometric validation of this language version was not conducted in the present study, and no adaptation-specific statistical indicators were calculated. Therefore, resilience scores should be interpreted with caution, and future studies should evaluate the reliability, validity, factor structure, and measurement invariance of the Ukrainian RS-25 in war-displaced populations. The generalizability of the findings is additionally constrained by the use of non-probability convenience sampling, recruitment in settings accessible to Ukrainian migrants and war-displaced persons, and the predominance of women in the study sample. Because no random sampling frame was available and participation depended on accessibility and willingness to take part in the study, selection bias cannot be excluded. Finally, the analyses were based on bivariate comparisons and did not include multivariable adjustment. As a result, the observed associations should be interpreted as unadjusted relationships and not as independent effects of resilience on health-related outcomes. Therefore, resilience should not be interpreted as a demonstrated protective factor in the present study, but rather as a resilience-related characteristic statistically associated with health-related self-assessments. Potential confounding factors, including age, gender, education, family situation, trauma exposure, length of stay in Poland, socioeconomic conditions, and access to healthcare, may have influenced both resilience and self-rated health. In particular, although all participants were included on the basis of war-related displacement, the study did not assess the intensity, type, or duration of war-related exposure or perceived war-related stress. These unmeasured factors may have influenced both resilience and health-related functioning. Future studies with larger samples should apply multivariable regression models to assess whether the observed associations remain significant after adjustment for these factors. In addition, the sample size calculation was based on a conservative population-based estimate rather than an a priori power calculation for association testing. Therefore, although the achieved sample size was sufficient for the exploratory objectives of the study, the analyses may have been underpowered to detect very small associations or to provide stable estimates for less frequent health problems. Notwithstanding these limitations, the study contributes to an underexplored area of refugee health research by focusing on an adult Ukrainian population living in a major receiving country (9, 23).

Future studies should extend this line of inquiry using longitudinal designs that can clarify the temporal relationship between resilience and health. It would also be valuable to distinguish between pre-migration trauma, peri-migration exposure, and post-migration living conditions, as these dimensions may affect resilience and self-rated health through different pathways. In addition, more nuanced analyses of resilience should consider both individual and contextual resources, including housing stability, employment, social support, language competence, and experiences of discrimination. Such work would help move the field beyond a narrow deficit-oriented perspective and toward a fuller understanding of adaptation in forcibly displaced populations (9, 26, 27).

In conclusion, this exploratory cross-sectional study found statistically significant unadjusted associations between resilience and self-rated health, physical functioning, physical activity, subjective wellbeing, and the presence of selected health problems among Ukrainian war-displaced adults in Poland. These results are consistent with the view that resilience is a relevant public health construct in refugee populations and suggest that adaptive resources should be considered in comprehensive health support for people affected by war and forced migration.

## Data Availability

The raw data supporting the conclusions of this article will be made available by the authors, without undue reservation.
