# Supplementary material for: Resilience and self-rated health among Ukrainian war-displaced adults in Poland: a cross-sectional study
Source: Front Public Health. 2026 May 29;14:1830164. doi: 10.3389/fpubh.2026.1830164 (PMC13259639; doi:10.3389/fpubh.2026.1830164)
Supplement: Supplementary file 1 [file Data_Sheet_1.docx]

**Supplementary Appendix S1**

**Author-designed health-related questionnaire**

**Note.** The Ukrainian version of this questionnaire was administered to participants. The English version below is provided for review and transparency purposes. The questionnaire was designed to collect sociodemographic information, self-reported health problems, and subjective ratings of selected health-related domains in two reference periods: formerly in Ukraine and currently in Poland.

1. **Ukrainian version**

**Інструкція для учасника/учасниці:** Будь ласка, прочитайте кожне запитання та оберіть відповідь, яка найкраще відповідає Вашій ситуації. У запитанні щодо проблем зі здоров’ям можна вибрати більше ніж одну відповідь, якщо це стосується Вашої ситуації. У частині щодо самооцінки здоров’я поставте оцінку від 1 до 5 окремо для періоду проживання в Україні та для поточного періоду проживання в Польщі.

# A. Соціодемографічна інформація

### 1. Стать

| ☐ Жінка | ☐ Чоловік |
| --- | --- |
| ☐ Небінарна особа | ☐ Віддаю перевагу не відповідати |

### 2. Вік

Вік у роках: ____________________

### 3. Рівень освіти

| ☐ Початкова освіта | ☐ Професійна освіта |
| --- | --- |
| ☐ Середня освіта | ☐ Вища освіта |

### 4. Сімейний стан

| ☐ Перебуваю у стосунках / шлюбі; мій партнер / моя партнерка проживає зі мною в Польщі |
| --- |
| ☐ Перебуваю у стосунках / шлюбі; мій партнер / моя партнерка проживає в Україні |
| ☐ Не перебуваю у стосунках / самотній(я) |

# B. Самооцінені проблеми зі здоров’ям

### 5. Будь ласка, вкажіть, чи маєте Ви такі проблеми зі здоров’ям

Можна вибрати більше ніж одну відповідь.

| ☐ Не маю жодних проблем зі здоров’ям | ☐ Захворювання серцево-судинної системи |
| --- | --- |
| ☐ Захворювання дихальної системи | ☐ Захворювання нервової системи |
| ☐ Захворювання травної системи | ☐ Захворювання опорно-рухового апарату |
| ☐ Проблеми з психічним здоров’ям |  |

# C. Самооцінка доменів функціонування, пов’язаних зі здоров’ям

### 6. Як би Ви оцінили наведені нижче аспекти під час проживання в Україні та у поточний період проживання в Польщі?

**Шкала відповідей:** 1 = дуже погано, 2 = погано, 3 = задовільно, 4 = добре, 5 = дуже добре.

| **Домен** | **Раніше в Україні (1–5)** | **Зараз у Польщі (1–5)** |
| --- | --- | --- |
| Ваш загальний стан здоров’я | ______ | ______ |
| Ваше самопочуття / добробут | ______ | ______ |
| Ваше фізичне функціонування | ______ | ______ |
| Ваша фізична активність | ______ | ______ |

**Короткі пояснення доменів**

**Загальний стан здоров’я:** Ваша загальна суб’єктивна оцінка власного здоров’я.

**Самопочуття / добробут:** Ваша суб’єктивна оцінка самопочуття та загального добробуту у повсякденному житті.

**Фізичне функціонування:** Ваша суб’єктивна оцінка здатності виконувати повсякденні фізичні дії та функціонувати фізично.

**Фізична активність:** Ваша суб’єктивна оцінка рівня рухової активності у повсякденному житті.

Таблиця відповідей

**II. English translation for review and transparency purposes**

**Instructions for respondents**

Please answer the following questions by selecting the response option that best applies to you. For the final question, please rate each domain separately for the period when you were living in Ukraine and for your current situation in Poland. Responses are anonymous and will be used only for research purposes.

**A. Sociodemographic information**

1. Gender

| ☐ Woman | ☐ Man |
| --- | --- |
| ☐ Non-binary | ☐ Prefer not to answer |

2. Age (in years): __________

3. Education level

| ☐ Primary education | ☐ Vocational education |
| --- | --- |
| ☐ Secondary education | ☐ Higher education |

4. Marital/relationship status

| ☐ In a relationship (partnered/married); my partner lives with me in Poland |
| --- |
| ☐ In a relationship (partnered/married); my partner lives in Ukraine |
| ☐ Single |

**B. Self-reported health problems**

5. Please select your main health problem. If more than one applies, select all that are relevant.

| ☐ I do not have any health problems | ☐ Cardiovascular disorders |
| --- | --- |
| ☐ Respiratory disorders | ☐ Nervous system disorders |
| ☐ Digestive system disorders | ☐ Musculoskeletal system disorders |
| ☐ Mental health problems |  |

**C. Self-rated health-related domains**

6. How would you rate the following domains during your time in Ukraine and currently in Poland? Please use the scale below:

**1 = very poor, 2 = poor, 3 = fair, 4 = good, 5 = very good**

| **Domain** | **Formerly in Ukraine (1–5)** | **Currently in Poland (1–5)** |
| --- | --- | --- |
| Overall health |  |  |
| Well-being |  |  |
| Physical functioning |  |  |
| Physical activity |  |  |

**Domain descriptions used for reporting purposes**

**Overall health:** the respondent’s global subjective appraisal of general health.

**Well-being:** the respondent’s subjective perception of well-being in daily life.

**Physical functioning:** the respondent’s perceived ability to perform everyday physical activities and bodily functioning.

**Physical activity:** the respondent’s subjective assessment of their level of movement or activity in daily life.
